# Presence of *Cryptosporidium parvum* in pre-washed vegetables from different supermarkets in South East England: A pilot study

**DOI:** 10.1007/s00436-024-08250-w

**Published:** 2024-06-01

**Authors:** Aisha Jamo Suleiman, Daphne E. Mavrides, Sadiya Maxamhud, Eleni Gentekaki, Anastasios D. Tsaousis

**Affiliations:** 1 Laboratory of Molecular and Evolutionary Parasitology, RAPID Group, School of Biosciences, University of Kent, Canterbury, Kent CT2 7NJ UK; 2 Department of Veterinary Medicine, University of Nicosia School of Veterinary Medicine, 2414 Nicosia, Cyprus; 3 Department of Basic and Clinical Sciences, University of Nicosia Medical School, Nicosia, Cyprus

**Keywords:** Cryptosporidium, Vegetables, Small subunit ribosomal RNA, Epidemiology, Food, Public health

## Abstract

*Cryptosporidium* is an important water-borne and food-borne parasite with a high burden of disease*.* This organism has been shown to contaminate various leafy vegetables; however, studies assessing the presence of *Cryptosporidium* spp in pre-washed and ready-to-eat vegetables are limited. Routine surveillance in the UK revealed a nationwide exceedance of human cases of *Cryptosporidium*. Therefore, this study aims to assess the presence of this parasite in pre-washed vegetables from supermarkets in the UK. A total of 36 samples were purchased from four different supermarkets. A nested PCR targeting the SSU rRNA was carried out on 24 samples, 58% were PCR-positive for *Cryptosporidium.* Sanger sequencing confirmed that, of these sequences, 4/24 (17%) produced significant similarities to *Cryptosporidium parvum*. This study provides evidence for the presence of *C. parvum* in pre-washed and ready-to-eat vegetables. Future work to identify the point of contamination is required.

**Supplementary Information:**

The online version contains supplementary material available at 10.1007/s00436-024-08250-w.

## Introduction

*Cryptosporidium* is an intracellular extracytoplasmic protozoan parasite that belongs to the phylum Apicomplexa (Ryan et al. 2016). It is the causative agent of cryptosporidiosis, which affects both humans and animals. Two species of *Cryptosporidium* most commonly cause the disease in humans and other vertebrate hosts: *C. parvum* and *C. hominis* (Widmer et al. 2020; Pinto et al. 2022). Symptoms in humans range from self-limiting nausea and abdominal cramps to life-threatening disease in children under 5 years of age and immunocompromised hosts, such as HIV-positive individuals (Wang et al. 2018). It is the second most common cause of diarrhoea in children under 12 months of age and the second most common cause of diarrhoea-associated deaths in children between 12 and 23 months (Wang et al. 2018; Khalil et al. 2018; Kotloff et al. 2013). The parasite is transmitted through the faecal-oral route typically acquired via ingestion of food or water contaminated with oocysts, the transmission form of the parasite. Studies also suggest that *Cryptosporidium* can also be transmitted through inhalation of oocysts (Pinto et al. 2022; Nyangulu et al. 2019).

In industrialized countries such as the USA and the UK, *Cryptosporidium* has been a cause of waterborne and foodborne outbreaks. In the UK, between 1992 and 2003, *Cryptosporidium* caused 67 outbreaks in drinking water and swimming pools with the main causes being *C. parvum* and *C. hominis* (Smith et al. 2006)*.* To date, the largest foodborne outbreak of the parasite reported in England and Scotland was from pre-cut vegetables sold in supermarkets with 74 confirmed lab cases of *C. parvum* (McKerr et al. 2015).

One of the reasons *Cryptosporidium* is prone to causing waterborne and foodborne outbreaks is due to its ability to form oocysts that resist environmental pressures. The formation of oocysts enables *Cryptosporidium* to survive outside a host for several months (Weir 2001). Moreover, the oocysts are resistant to disinfection with chlorine and can only be reliably removed by boiling water or filtration (Weir 2001).

More recently, a spike in *Cryptosporidium* cases was noted during routine surveillance between August and October 2023 across various regions of the UK with about 2411 laboratory-confirmed cases (Peake et al. 2023). Current data shows *C. hominis* accounted for most of the cases and was associated with exposure to foreign travel, swimming, farm animals, and food consumption (Peake et al. 2023). However, no single exposure accounts for the widespread exceedance in cases, and investigations are ongoing. In recent years, ready-to-eat (RTE) vegetables have become increasingly attractive to consumers by readily providing healthy, no-preparation-needed nutrition. However, concerns have been raised regarding the detection of microorganisms such as bacteria, viruses, fungi, and parasites in RTE vegetables (Zhang et al. 2020; Gizzie and Adukwu 2023). In light of this, we wanted to investigate pre-washed and RTE vegetables as a means for *Cryptosporidium* spreading. Hence, this pilot study aims to assess the presence of *Cryptosporidium* contamination in RTE from 36 prepacked bags containing a variety of vegetables (Table 1) from different supermarkets in the UK.

**Table 1:** Component ingredients in RTE samples. Nested PCR samples positive for *Cryptosporidium* spp. with *SSU* rRNA and *gp60* sequencing results. N/A (Not available)

Supermarket	Sample ID	Component vegetables	Country of origin/place of packaging	*Cryptosporidium gp60*
A	Mild and tender mixed baby leaf (MT1)	Baby spinach, green butterhead lettuce, red chard, ruby red chard	N/A / UK	
A	Mild and tender mixed baby leaf (MT2)	Baby spinach, green butterhead lettuce, red chard, ruby red chard	N/A / UK	
B	Sweet leaf salad (SLS1)	Iceberg lettuce, carrot, romaine lettuce, white cabbage	UK / UK	
B	Sweet leaf salad (SLS2)	Iceberg lettuce, carrot, romaine lettuce, white cabbage	UK / UK	
C	Sweet and crunchy salad (SCS1)	Iceberg lettuce, red cabbage, carrot	N/A	
C	Sweet and crunchy salad (SCS2)	Iceberg lettuce, red cabbage, carrot	N/A	*C. parvum*
C	Beetroot salad (BRS1)	Spinach, chard, lamb’s lettuce	N/A	*C. parvum*
C	Beetroot salad (BRS2)	Spinach, chard, lamb’s lettuce	N/A	*C. parvum*
D	British butterhead salad (BBS1)	Red butterhead lettuce, green butterhead lettuce, baby spinach	UK / UK	
D	British butterhead salad (BBS2)	Red butterhead lettuce, green butterhead lettuce, baby spinach	UK / UK	*C. parvum* IIaA17G2R1
D	Rocket and baby leaf salad (RBS1)	Rocket, baby red leaves, baby spinach, mizuna	A mix of UK and non-UK produce	
D	Rocket and baby leaf salad (RBS2)	Rocket, baby red leaves, baby spinach, mizuna	A mix of UK and non-UK produce	
D	British pea shoot salad (BPS1)	Baby spinach, pea shoots, baby red leaves	UK / UK	
D	Bistro salad (BS2)	Shredded beetroot, baby spinach, lambs’ lettuce, ruby red chard	A mix of UK and non-UK produce	

## Materials and methods

In the period between May 2023 and July 2023, a total of 36 pre-washed vegetables were purchased from four major supermarkets (A, B, C, D) in Canterbury, Kent County, UK. Samples were randomly chosen from packaged RTE vegetables and duplicates from each variety were obtained. Two hundred and fifty grams of vegetables per package were placed in a beaker with 0.9% NaCl and thereafter placed in a shaking incubator at 37 °C for 60 min. The resultant fluid was collected into 50-ml falcon tubes and centrifuged at 3000 rcf for 10 min at 4 °C. The supernatant was then discarded, and pellets were frozen at − 20 °C or used immediately for DNA extraction as previously described (Jinatham et al. 2023). Genomic DNA was extracted from all samples using Purelink™ Microbiome DNA Purification Kit (ThermoFisher Scientific, UK) (soil samples) according to the manufacturer’s instructions. A nested PCR was done on the extracted DNA to amplify the small subunit (*SSU*) rRNA gene sequence (Supplementary information). The 60-kDa glycoprotein (*gp60)* gene was also amplified by nested PCR on samples that were PCR-positive for *SSU* rRNA (Supplementary information) (Pinto et al. 2021). Gel extraction of the amplified products was carried out using a QIAquick Gel Extraction Kit (cat. Nos. 28704 and 28,706) according to the manufacturer’s protocol. Purified products were sent off for Sanger sequencing at Eurofins Genomics (Cologne, Germany). The obtained chromatograms were viewed and edited using the Snap Gene Viewer software. Ambiguous nucleotides at the ends of each read were removed. We used nucleotide sequences as queries to Basic Local Alignment Search Tool (BLASTn) (https://blast.ncbi.nlm.nih.gov/Blast.cgi) against the nr database in GenBank. Cloning was performed on sequences of unclear chromatographs as previously described (Betts et al. 2020).

## Results

Twelve out of 36 samples did not yield sufficient DNA (less than 0.01 ng/μl) and were excluded from further analyses. Of the remaining 24, 14 were PCR-positive using *SSU* rRNA gene sequence amplification by nested PCR. All 14 were genotyped using gp60. Finally, a total of 4/24 were sequence positive for *Cryptosporidium*: BBS2, BRS1, BRS2 and SCS2. The percent identity was 99.28% (CP082114), 99.16% (CP141123), 97.05% (CP141123) and 94.60% (MK391454), respectively.

The sequences have been submitted to GenBank under accession numbers PP502149, PP828699-PP828701.

## Discussion

Infection with *Cryptosporidium* causes long-term sequelae such as diarrhea, abdominal pain, nausea, headaches, and fatigue. Infection is associated with failure to thrive, malnutrition, cognitive deficits, and stunting in infants and children (Khalil et al 2018; Vanathy et al. 2017). Therefore, efforts to prevent infection and outbreaks should be strictly enforced. In Western countries, one of the sources of infection that is frequently overlooked is the consumption of RTE vegetables. The Food Standards Agency (FSA) in the UK has recommended rewashing RTE (ACM/891 n.d.). The findings of our study could have implications for public health in the UK. Notably, the species identified herein was the zoonotic *C. parvum* rather than the anthroponotic *C. hominis* as is the case with the current exceedance. As such, further investigation is warranted.

Pre-washed and ready-to-eat vegetables undergo more thorough washing with water containing chlorine disinfectants as compared to unpackaged vegetables and are expected to be free from parasites and therefore, can be eaten straight from the packaging. However, due to the resistance of oocysts to chlorine disinfection and their persistence on the vegetable surface, some remain, and are likely to cause illness (Barlaam et al. 2022). This is in keeping with previous evidence of *Cryptosporidium* contamination in pre-washed and ready-to-eat vegetables. Dixon et al. identified *Cryptosporidium parvum* in 5.9% of RTE vegetables in Canada (Dixon et al. 2013), in line with the results herein. Another study identified *Cryptosporidium* as the second most prevalent protozoan parasite present in 0.9% of RTE vegetables in Italy (Caradonna et al. 2017). As Italy is the second largest supplier of vegetables in Europe (Caradonna et al. 2017), it would be assumed that any contamination arising from production could lead to widespread distribution throughout Europe.

Herein, the parasite could originate from external contamination of vegetables happening along the chain of production including oocyst present in irrigation water, fertilizers, or transfer by handlers at the point of harvesting, processing, packaging, and/or transportation (Barlaam et al. 2022). Deciphering the point in the chain where contamination occurred is further complicated by the presence of multiple vegetable varieties in a package. Moreover, mixing contaminated and non-contaminated vegetables and recycling wash water could all lead to cross-contamination (Koutsoumanis et al. 2018). The presence of *Cryptosporidium* in pre-washed vegetables could mean revisiting the sanitation methods employed by suppliers along the chain of production such as improved hygiene measures during harvesting, processing, packaging, transportation, and storage (Jung et al. 2014). Other methods of sanitizing irrigation and washing water such as filtration, boiling, and the use of ozone could be explored (Gérard et al. 2019). There is also a need for increased awareness among consumers on adequate storage of vegetables and handwashing before eating. Better monitoring and regulation of pre-washed vegetables by appropriate authorities should also be encouraged.

This pilot study could unlock a potential common source of *Cryptosporidium* infection across the various regions in the UK. The vegetables from individual chain supermarkets are packaged in their own central facilities and subsequently distributed nationwide. Our study could contribute to developing better strategies to prevent infection with *Cryptosporidium.* Further studies could focus on tracking the exact point of contamination and addressing it.

### Supplementary Information

Below is the link to the electronic supplementary material.

Supplementary file1 (DOCX 24 KB)

## Data Availability

The sequences have been submitted to GenBank under accession numbers PP502149, PP828699-PP828701.

## References

[CR1] ACM/891 (n.d.) Advisory Committee on the Microbiological Safety of Food Fresh produce: Agency advice on re-washing ready-to-eat leafy salads. UK Food standards Agency. Issue (cited 2024 Feb 25). Available from: http://www.food.gov.uk/multimedia/pdfs/ecolitaskfinreport.pdf

[CR2] Barlaam A, Sannella AR, Ferrari N, Temesgen TT, Rinaldi L, Normanno G et al (2022) Ready-to-eat salads and berry fruits purchased in Italy contaminated by *Cryptosporidium* spp., *Giardia duodenalis*, and *Entamoeba histolytica*. Int J Food Microbiol, vol 370. p 109634 10.1016/j.ijfoodmicro.2022.109634 35316671

[CR3] Betts EL, Gentekaki E, Tsaousis AD (2020). Exploring micro-eukaryotic diversity in the gut: co-occurrence of Blastocystis subtypes and other protists in zoo animals. Front Microbiol.

[CR4] Caradonna T, Marangi M, Del Chierico F, Ferrari N, Reddel S, Bracaglia G (2017). Detection and prevalence of protozoan parasites in ready-to-eat packaged salads on sale in Italy. Food Microbiol.

[CR5] Dixon B, Parrington L, Cook A, Pollari F, Farber J (2013). Detection of *Cyclospora*, *Cryptosporidium*, and *Giardia* in ready-to-eat packaged leafy greens in Ontario Canada. J Food Prot.

[CR6] EFSA Panel on Biological Hazards (BIOHAZ), Koutsoumanis K, Allende A, Alvarez-Ordóñez A, Bolton D, Bover-Cid S et al (2018) Public health risks associated with food-borne parasites. E F S A Journal. 16(12):e05495. 10.2903/j.efsa.2018.5495 10.2903/j.efsa.2018.5495 PMC7009631 32625781

[CR7] Gérard C, Franssen F, La Carbona S, Monteiro S, Cozma-Petruţ A, Utaaker KS (2019). Inactivation of parasite transmission stages: efficacy of treatments on foods of non-animal origin. Trends Food Sci Technol.

[CR8] Gizzie NY, Adukwu E (2023) Assessment of microbial quality and effect of rinsing on pre-packaged salads. (cited 2023 Aug 25) 10.13140/RG.2.1.1525.2561

[CR9] Jinatham V, Wandee T, Nonebudsri C, Popluechai S, Tsaousis AD, Gentekaki E (2023) Blastocystis subtypes in raw vegetables from street markets in northern Thailand. Parasitol Res 122(4):1027–31 (cited 2024 Feb 25) 10.1007/s00436-023-07781-y 36658225

[CR10] Jung Y, Jang H, Matthews KR (2014). Effect of the food production chain from farm practices to vegetable processing on outbreak incidence. Microb Biotechnol.

[CR11] Khalil IA, Troeger C, Rao PC, Blacker BF, Brown A, Brewer TG (2018). Morbidity, mortality, and long-term consequences associated with diarrhoea from *Cryptosporidium* infection in children younger than 5 years: a meta-analyses study. Lancet Glob Health.

[CR12] Kotloff KL, Nataro JP, Blackwelder WC, Nasrin D, Farag TH, Panchalingam S (2013). Burden and aetiology of diarrhoeal disease in infants and young children in developing countries (the Global Enteric Multicenter Study, GEMS): a prospective, case-control study. Lancet.

[CR13] McKerr C, Adak GK, Nichols G, Gorton R, Chalmers RM, Kafatos G, Cosford P, Charlett A, Reacher M, Pollock KG, Alexander CL, Morton S (2015) An Outbreak of *Cryptosporidium parvum* across England & Scotland Associated with Consumption of Fresh Pre-Cut Salad Leaves. PLoS One. 10(5):e0125955. 10.1371/journal.pone.0125955 10.1371/journal.pone.0125955 PMC4446264 26017538

[CR14] Nyangulu W, Van Voorhis W, Iroh Tam PY (2019). Evaluating respiratory cryptosporidiosis in pediatric diarrheal disease: protocol for a prospective, observational study in Malawi. BMC Infect Dis.

[CR15] Peake L, Inns T, Jarvis C, King G, Rabie H, Henderson J et al (2023) Preliminary investigation of a significant national *Cryptosporidium* exceedance in the United Kingdom, August 2023, and ongoing. Eurosurveillance 28(43):2300538 (cited 2023 Nov 26) 10.2807/1560-7917.ES.2023.28.43.2300538 PMC10604540 37883039

[CR16] Pinto P, Ribeiro CA, Hoque S, Hammouma O, Leruste H, Détriché S (2021). Cross-border investigations on the prevalence and transmission dynamics of *Cryptosporidium* species in dairy cattle farms in western mainland Europe. Microorganisms.

[CR17] Pinto P, Ribeiro CA, Kváč M, Tsaousis AD (2022) *Cryptosporidium*. [cited 2023 Aug 5];331–89. Available from: https://link.springer.com/chapter/10.1007/978-3-030-80682-8_7

[CR18] Ryan U, Paparini A, Monis P, Hijjawi N (2016). It’s official – *Cryptosporidium* is a gregarine: what are the implications for the water industry?. Water Res.

[CR19] Smith A, Reacher M, Smerdon W, Adak GK, Nichols G, Chalmers RM (2006). Outbreaks of waterborne infectious intestinal disease in England and Wales, 1992–2003. Epidemiol Infect.

[CR20] Vanathy K, Parija S, Mandal J, Hamide A, Krishnamurthy S (2017). Cryptosporidiosis: a mini-review. Trop Parasitol.

[CR21] Wang ZD, Liu Q, Liu HH, Li S, Zhang L, Zhao YK (2018). Prevalence of *Cryptosporidium*, microsporidia and *Isospora* infection in HIV-infected people: a global systematic review and meta-analysis. Parasit Vectors.

[CR22] Weir E (2001). Public Health: The cryptic nature of cryptosporidiosis. CMAJ: Can Med Assoc J.

[CR23] Widmer G, Köster PC, Carmena D (2020). *Cryptosporidium hominis* infections in non-human animal species: revisiting the concept of host specificity. Int J Parasitol.

[CR24] Zhang H, Yamamoto E, Murphy J, Locas A (2020). Microbiological safety of ready-to-eat fresh-cut fruits and vegetables sold on the Canadian retail market. Int J Food Microbiol.

