# Supplementary material for: Presence of Cryptosporidium parvum in pre-washed vegetables from different supermarkets in South East England: A pilot study
Source: Parasitol Res. 2024 Jun 1;123(6):230. doi: 10.1007/s00436-024-08250-w (PMC11142934; doi:10.1007/s00436-024-08250-w)
Supplement: Supplementary file 1 — Supplementary file1 (DOCX 24 KB) [file 436_2024_8250_MOESM1_ESM.docx]

**Table S1.** PCR primers for Cryptosporidium used in the study.

| **Gene sequence amplified** | **Reactions** | **Primers** | **Sequence 5'-3'** | **Amplified fragment** | **Reference** |
| --- | --- | --- | --- | --- | --- |
| *SSU* rRNA | 1^st^ | Forward (CRY_SSU_F1)  Reverse (CRY_SSU_R1) | GATTAAGCCATGCATGTCTAA  TTCCATGCTGGAGTATTCAAG | 732 bp | (17) |
|  | 2^nd^ | Forward (CRY_SSU_F2)  Reverse (CRY_SSU_R2) | CAGTTATAGTTTACTTGATAATC    CCTGCTTTAAGCACTCTAATTTTC | 631 bp |  |
| *gp60* | 1^st^ | AL3531  AL3535 | ATAGTCTCCGCTGTATTC  GGAAGGAACGATGTATCT | 1000 bp | (17) |
|  | 2^nd^ | AL3532  AL3534 | TCCGCTGTATTCTCAGCC  GCAGAGGAACCAGCATC | 850 bp |  |
